# Mulberry Leaf Extract Attenuates Oxidative Stress-Mediated Testosterone Depletion in Streptozotocin-Induced Diabetic Rats

**Published:** 2014-03

**Authors:** Mohammad Reza Hajizadeh, Ebrahim Eftekhar, Fatemeh Zal, Aida Jafarian, Zohreh Mostafavi-Pour

**Affiliations:** 1 Department of Biochemistry, Rafsanjan University of Medical Sciences, Rafsanjan, Iran;; 2 Molecular Medicine Research Center, Department of Biochemistry, Hormozgan University of Medical Sciences, Bandar Abbas, Iran;; 3 Department of Reproductive Biology, School of Advanced Medical Sciences and Technologies, Shiraz University of Medical Sciences, Shiraz, Iran;; 4 Recombinant Protein Laboratory, Department of Biochemistry, School of Medicine, Shiraz University of Medical Sciences, Shiraz, Iran;; 5 Maternal-Fetal Medicine Research Center, Hafez Hospital, Shiraz University of Medical Sciences, Shiraz, Iran

**Keywords:** Diabetes mellitus, Morus alba, Oxidative stress, Testosterone

## Abstract

**Background: **It has been proposed that oxidative stress may contribute to the development of testicular abnormalities in diabetes. Morus alba leaf extract (MAE) has hypoglycemic and antioxidant properties. We, therefore, explored the impact of the administration of MAE on steroidogenesis in diabetic rats.

**Methods: **To address this hypothesis, we measured the serum level of glucose, insulin, and free testosterone (Ts) as well as oxidative stress parameters (including glutathione peroxidase, glutathione reductase, total antioxidant capacity, and malondialdehyde) in the testis of control, untreated and MAE-treated (1 g/day/kg) diabetic rats. In order to determine the likely mechanism of MAE action on Ts levels, we analyzed the quantitative mRNA expression level of the two key steroidogenic proteins, namely steroid acute regulatory protein (StAR) and P450 cholesterol side-chain cleavage enzyme (P450scc), by real-time PCR.

**Results:** The MAE-treated diabetic rats had significantly decreased glucose levels and on the other hand increased insulin and free Ts levels than the untreated diabetic rats. In addition, the administration of MAE to the diabetic rats restored the oxidative stress parameters toward control. Induction of diabetes decreased testicular StAR mRNA expression by 66% and MAE treatment enhanced mRNA expression to the same level of the control group. However, the expression of P540scc was not significantly decreased in the diabetic group as compared to the control group.

**Conclusion:** Our findings indicated that MAE significantly increased Ts production in the diabetic rats, probably through the induction of StAR mRNA expression levels. Administration of MAE to experimental models of diabetes can effectively attenuate oxidative stress-mediated testosterone depletion.

## Introduction

Diabetes mellitus (DM) is one of the most common chronic diseases worldwide. It has been estimated that the prevalence of DM will increase from 275 million adults in 2010 to 439 million by 2030.^1^ It has been posited that oxidative stress plays a pivotal role in the pathogeneses of DM.^2^ Accumulated data suggest that DM is linked with male reproductive dysfunction.^3^ In rats, diabetes induces apoptosis in the testicular germ cell^4^ and decreases sperm count and plasma testosterone (Ts) levels.^5^ However, the potential contribution of oxidative stress due to diabetic condition in the development of testicular abnormalities has not been fully clarified. It has been shown that reactive oxygen species (ROS) can inhibit the steroid hormone production of cultured leydig cells by directly affecting the steroidogenic enzymes.^6^ Be that as it may, the testicular activity of steroidogenic proteins under diabetic condition has yet to be studied. Steroid acute regulatory protein (StAR) and P450 cholesterol side-chain cleavage enzyme (P450scc) are two important proteins that catalyze the first steps in steroidogenesis.^7^ Diemer et al.^8^ demonstrated that the in vitro exposure of MA-10 tumor leydig cells to ROS decreased StAR protein expression levels.

Morus alba (mulberry) is a plant rich in phytochemicals, which have an important role in diet-based therapies to cure several diseases.^9^ Antioxidant and antidiabetic activity of Morus alba leaf extract (MAE) in Streptozotocin-induced diabetic rats has been shown.^10^^,^^11^

The objective of this study was to investigate the effect of the long-term administration of MAE on oxidative stress markers and steroidogenesis in diabetic rats. The likely mechanism of MAE action on steroidogenesis was also explored.

## Materials and Methods

*Chemicals*

Streptozotocin, thiobarbituric acid (TBA), 1,1,3,3-tetramethoxypropane, reduced glutathione (GSH), oxidized glutathione (GSSG), nicotine-amid-adenine-dinucleotide phosphate (NADPH), glutathione reductase (GR), tripyridyl-s-triazine (TPTZ), and other high-grade chemicals were purchased from Sigma Chemical Company (St. Louis, Missouri, USA).

*Preparation of Morus Alba Leaf Extract*

Leaves of Morus alba were collected from a local area (Shiraz, Iran) in April. Having been authenticated by the Botany Department of Shiraz University, dried leaves were extracted with 50% of ethanol. The mixture was filtered, concentrated in a vacuum evaporator, and then lyophilized. Using this procedure, the yield was 22% of the starting dry weight of the leaves. The final extract was kept in an air-tight container at -80°C until it was used.

*Induction of Diabetes*

Diabetes was induced by a single intraperitoneal injection of 50 mg/kg body weight Streptozotocin (STZ) dissolved in 0.1 M citrate buffer (pH 4.5). Seven days after STZ injection, the blood was collected from the tail vein to determine the fasting blood glucose level. Rats with blood glucose >300 mg/dl were considered diabetic. The experiment was carried out 5 days after the confirmation of the existence of diabetes mellitus. 

*Experimental Design*

Thirty healthy adult male Wistar rats (8 weeks old), weighting about 250±10 g, were obtained from the Animal House Unit of Shiraz University of Medical Sciences, Shiraz, Iran. They were kept under controlled conditions with a regular light cycle (12; 12 h light; dark cycle). During the experiment, the rats had free access to food and water. All the animal experiments were approved by the Ethics Committee of Shiraz University of Medical Sciences. Using a random number table, the rats were divided into three groups with 10 rats in each group and were treated through a gavage tube for a period of 2 months as follows: 1) non-diabetic rats with distilled water (control); 2) diabetic rats with distilled water; and 3) diabetic rats with MAE extract at the dose of 1 g/kg per day. At the end of the 8^th^ week, all the rats were euthanized by an overdose of anesthetic drug. Blood samples were collected into a tube and serum was separated for the determination of glucose, insulin, and free Ts. The testes were separated and washed with ice cold 0.9% NaCl, blotted dry, decapsulated, and homogenized at 4°C in 50 mM phosphate buffer saline (pH 7.4). Homogenate was centrifuged at 10,000×g for 10 min at 4°C, and the supernatant was used to measure GR and glutathione peroxidase (GPx) activities and malondialdehyde (MDA) levels as well as total antioxidant capacity (TAC). The total protein content of the supernatant was determined using the Bradford method.^12^

*Measurement of Serum Glucose, Insulin, and Free Ts*

Serum glucose level was measured using a commercial kit (Pars Azmoon Company, Tehran, Iran) based on the hexokinase method. Serum insulin and free Ts concentrations were measured using the enzyme linked immunosorbent assay (ELISA) kit (DRG instruments, GmbH, Marburg, Germany).  

*Biochemical Analysis of Testicular Antioxidant Status*

MDA, an indirect index of lipid peroxidation, was assayed as TBARS (thiobarbituric reactive substance) using a colorimetric method.^13^TBARS concentration was calculated using 1,1,3,3-tetramethoxypropane as a standard. The results are expressed as nanomoles per mg protein.

GPx was measured as described by a previous study.^14^ Briefly, the enzymatic reaction in the cuvette that contained NADPH, GSH, NaN_3_,and GR was initiated by adding H_2_O_2_ and the change in absorbance was monitored spectrophotometrically at 340 nm.^14^

GR was assayed using the method described by Carlberg and Mannervik.^15^ The GR assay was performed in a tube that contained Tris–HCl buffer, EDTA, GSSG, NADPH, and a sample in a final volume of 1.0 mL. The decrease in the absorbance of NADPH at 340 nm was monitored using a spectrophotometer.^15^

The results are expressed as units of enzyme activities per mg of protein.

TAC was determined by the ferric-reducing antioxidant power assay (FRAP assay).^16^

*RNA Extraction and Real-Time RT-PCR Analysis*

Total RNA was extracted from the testis using Tripure RNA Isolation Reagent according to the manufacturer’s instructions (Roche, Germany) and was quantified by spectrophotometry. The integrity of the extracted total RNA was checked by agarose gel electrophoresis and verified by the presence of 28S, 18S rRNA bands. Total RNA was reverse transcribed into the first strand of complementary DNA (cDNA) with Revert AidTM First Strand cDNA Synthesis Kit (Fermentas, EU).

Real-time RT-PCR assays for the quantitative determination of StAR, P450scc, and beta actin (internal control) were performed in duplicate using the ABI system (Applied ABI Company, Foster City, CA USA). Primer sequences which were used for the beta actin, StAR, and P450scc amplification are shown in table 1. Amplifications were performed in 25-μl mixtures containing 1/20 volume of cDNA preparation (2 μl) and 1X SYBR Green PCR Master Mix (PE Applied Biosystems, CA, USA) according to the manufacturer’s instructions. Additionally, cDNA samples were amplified with pre-cycling heat activation at 95°C for 10 min, followed by 40 cycles (15 s at 95°C, 30 s at 58°C, and 30s at 60°C). The concentration of beta actin was determined in each sample, and the relative quantification of mRNA in each sample was conducted using the comparative Ct (threshold cycle) method. The results are depicted as mRNA copies/beta actin units, allowing a direct comparison of the expression levels between the different mRNA species. The quality and correct size of the PCR products were checked by electrophoresis on 1% agarose gel.

**Table 1:** Sequence of the primers used for real-time PCR

**Genes**	**Forward**	**Reverse**	**Product size**
Actin	CCACACCCGCCACCAGTTCG	CTAGGGCGGCCCACGATGGA	138
P450scc	AAACACCACGCACTTCCG	TAGGCTTCTCAGGCATCAGG	185
StAR	CTGCTAGACCAGCCCATGGAC	TGATTTCCTTGACATTTGGGTTCC	91

*Statistical Analysis*

The data are expressed as mean±SEM. Differences in the variables between the three groups were determined by the analysis of variance (ANOVA), followed by the Student Newman-Kelus test, to show specific group differences. The level of significance was taken as P<0.05. All the statistical analyses were carried out using SPSS software (SPSS for Windows, version 12.0).

## Results

*Effect of MAE on Body Weight, Serum Glucose, Insulin, and Free Ts*

The mean value of body weight, serum glucose, insulin, and free Ts level in the studied groups are shown in table 2. There was a reduction in body weight by 45% in the diabetic rats as compared to the control rats. The administration of MAE to the diabetic rats increased their body weight by only 12%, when compared to the untreated animals.

**Table 2:** Body weight and serum levels of glucose, insulin, and free testosterone in the three study groups

**Groups**	**Body weight (g)**	**Glucose (mg/dl)**	**Insulin (pmol/l)**	**Free testosterone (pg/ml)**
Control	328.5±3.3	110.3±2.5	677.2±2.5	3.4±0.5
Diabetic	178.1±2.9^a^	437.2±10.2^a^	260.2±1.7^a^	0.5±0.05^a^
MAE treated diabetic	202.2±2.8^b^	322.4±25.6^b^	386.6±3.2^b^	1.3±0.2^b^

Data are mean±SEM of 10 rats in each group. **^a^**Significant as compared with the control group, P<0.05; **^b^**Significant as compared with the diabetic group, P<0.05

Eight weeks administration of MAE (1 g/kg/day) to the diabetic rats significantly reduced the glucose level (26%; P=0.008). However, this value was still higher than that of the control group. The MAE-treated diabetic rats had significantly higher insulin and free Ts levels as compared with the diabetic group (32% and 61%, respectively; P=0.03). 

*Effect of MAE on Oxidative Stress Parameters*

Table 3 shows the mean values of oxidative stress parameters, including GPx, GR, TAC, and MDA in the testes of the control, diabetic, and MAE-treated diabetic rats.

The MAE-treated diabetic rats had significantly lower MDA levels as compared with the diabetic group (35%; P=0.02). TAC, GPx, and GR activities in the testes of the diabetic rats were significantly lower than those of the control group (41%, 33%, and 32%, respectively; P=0.04). MAE treatment noticeably increased these three oxidative stress parameters and normalized them to control level (table 3).

**Table 3:** Oxidative stress parameters in the three study groups

**Groups**	**MDA** **(nmol/mg protein)**	**GPx** **(U/mg protein)**	**GR** **(U/mg protein)**	**TAC** **(mmol/mg protein)**
Control	6.9±0.12	138±1.4	20.2±0.5	1.03±0.02
Diabetic	12.1±0.21^a^	89.2±1.5^a^	13.2±0.3^a^	0.63±0.01^a^
MAE-treated diabetic	7.8±0.15^b^	139.3±1.1^b^	20.7±0.3^b^	0.91±0.01^b^

Data are mean±SEM of 10 rats in each group. **^a^**Significant as compared with the control group, P<0.05; **^b^**Significant as compared with the diabetic group, P<0.05; MDA: malondialdehyde; GPx: glutathione peroxidase; GR: glutathione reductase; TAC: total antioxidant capacity

*Effect of MAE on mRNA Expression Level of StAR and P450scc*

**Figure 1:**
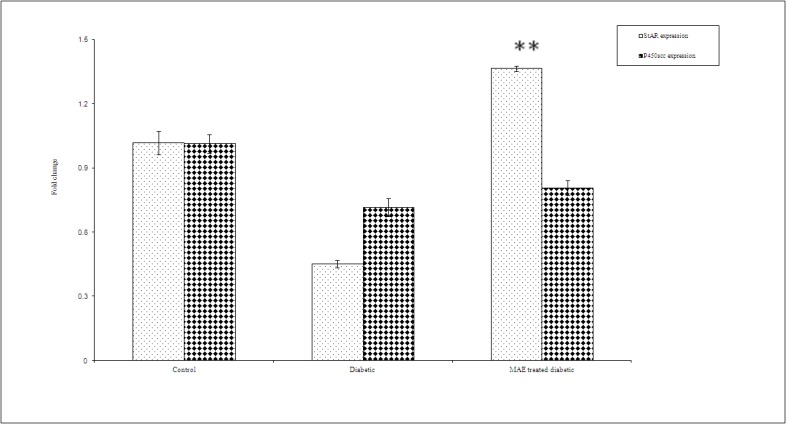
mRNA expression level of StAR and P450scc in the testis of the three study groups. Data are mean±SEM of 10 rats in each group. **Significant as compared with the diabetic group, P<0.05

The real-time PCR assays revealed single bands, corresponding to the expected product sizes of cDNAs for StAR (91 bp), P450scc (185 bp), and beta actin (138 bp). The specificity of the reactions was checked by melt curve analysis. Figure 1 presents the mean values of the testicular mRNA levels for StAR and P450scc in the control and diabetic rats. The untreated diabetic rats expressed lower levels of testicular StAR and P450scc mRNA as compared to the control group (66 % and 20%, respectively). However, a statistically significant reduction was observed only in StAR expression (P=0.03). It is interesting that treatment with 1 g/kg/day MAE significantly increased the StAR mRNA expression levels in the diabetic rats to control level.

## Discussion

The major findings of the present study were a marked reduction in the serum glucose level and measures of oxidative stress as well as an increase in the serum insulin, free Ts, and mRNA expression levels of StAR after 2 months treatment of diabetic rats with 1 g/kg/day MAE. 

It has been suggested that the hypoglycemic effect of MAE is induced via the inhibition of α-glucosidase by its active compound, 1-deoxynojirimycin.^17^ However, in our study, the hypoglycemic effect of MAE could be related to its insulinotropic property. In the MAE-treated diabetic rats, insulin showed a significantly higher level (33%) than in the untreated diabetes. In agreement with this result, Singub et al.^18^ showed that the oral administration of Egyptian Morus alba root bark for 10 days (0.6 g/kg/day) markedly increased the insulin level in STZ-induced diabetic rats. Nonetheless, the insulin stimulatory effect of MAE could not be observed in cell culture using insulinema cell line.^19^

In the present study, reduced GPx and GR activities and TAC as well as enhanced MDA levels clearly confirm the presence of high testicular oxidative stress in diabetes. Consistent with our results, a significant reduction in GPx and GR activities in the testis of diabetic rats has been reported by other researchers.^20^^,^^21^ However, the level of TAC was not evaluated in the previous studies.

Our results demonstrated that the administration of MAE to diabetic rats restored TAC and enzymes activities and decreased MDA levels. These effects of MAE are probably due to its hypoglycemic and free radical scavenging properties. MAE contains several compounds such as flavonoids glycoside that can act as a potent antioxidant.^22^

The role of oxidative stress in the development of testicular dysfunction under diabetic condition is not well understood. Unlüçerçi et al.^23^ found that oxidative stress was not involved in possible testicular complication of diabetes. In contrast, the results of a study by Shrilatha et al.^24^ suggested that oxidative stress might contribute to the induction of testicular dysfunction and reduced fertility of diabetic animals. In the present study, we observed significantly lower levels of free Ts in the diabetic rats. MAE treatment increased free Ts levels by 61% as compared to the diabetic rats. Nevertheless, this value was still lower than that in the control group. In the previous studies, a drop in total Ts was reported in diabetic rats.^20^^,^^25^ Since the concentration of total Ts depends on the serum level of albumin and sex-hormone binding globulins, measuring free Ts is a more sensitive indicator of Ts status. To the best of our knowledge, there is no report on the androgenic activity of MAE in the literature. In order to determine the likely mechanism of MAE action on Ts levels, we analyzed the mRNA expression level of two key steroidogenic proteins, namely StAR and P450scc. StAR is a protein that mediates the rate-limiting step in all steroid production. StAR participates in the transport of substrate cholesterol from the mitochondrial outer membrane to the inner membrane, where P450scc is located. The first committed step in the synthesis of steroid hormones is the conversion of cholesterol to pregnenolone, catalyzed by P450scc.^7^ Our results showed that the expression of StAR gene was significantly (60%) decreased in the diabetic group in comparison with the control. The mRNA expression of P450scc was also decreased, but it was not statistically significant. The MAE-treated diabetic rats showed induction of both mRNA levels, but a marked increase was only observed for StAR. It has been shown that oxidative stress can damage the key molecules of steroidogenic pathway, including StAR and cytochrome P450 enzymes in rat leydig cell culture.^8^^,^^26^ Other investigators have demonstrated that hydrogen peroxide can act directly on rat leydig cells and reduced Ts production through the inhibition of P450scc activity and StAR protein expression.^27^In our *in vivo* study, the reduced free Ts level in the diabetic rats could be related to oxidative stress (as shown by a decrease in TAC, GPx, and GR activities and increase in MDA levels) and induced downregulation of testicular StAR and P450scc mRNA levels. Our results demonstrated that StAR gene expression was more vulnerable to oxidative stress damage than P450scc (66% vs. 20% decrease in mRNA expression, respectively). The diminished expression of StAR results in decreased substrate cholesterol availability for steroidogenesis. The treatment of the diabetic rats with MAE markedly increased the mRNA level of StAR and thereafter Ts production. The antioxidant and free radical scavenging activity of MAE as indicated in this study may decrease the inhibitory and genotoxic effect of oxidative stress on StAR gene expression. It has been reported that elevated levels of ROS and lipid peroxidation may be involved in the reduced steroidogenic potency of cultured rat leydig cells.^28^ Our findings confirm and expand the role of oxidative stress in the development of testicular complication under diabetic condition.

## Conclusion

Our results indicated that MAE had not only hypoglycemic and antioxidant activities but also androgenic properties in the diabetic rats. The androgenic activity of MAE is probably due to the upregulation of StAR gene expression. The administration of MAE to experimental models of diabetes can effectively attenuate oxidative stress-mediated testosterone depletion.
